# Benchmarking Ploidy Estimation Methods for Bulk and Single‐Cell Whole Genome Sequencing

**DOI:** 10.1002/advs.202507839

**Published:** 2025-09-08

**Authors:** Yawei Song, Zilv Mei, Qijie Zheng, Qingqing Yuan, Yu Liang, Jiaqi Gao, Lang Zhou, Shuheng Wu, Wei Wu

**Affiliations:** ^1^ Key Laboratory of Multi‐Cell Systems Shanghai Institute of Biochemistry and Cell Biology Center for Excellence in Molecular Cell Science Chinese Academy of Sciences University of Chinese Academy of Sciences Shanghai 200031 China

**Keywords:** aneuploidy, ploidy estimation, single‐cell DNA sequencing, tumor purity, whole genome sequencing

## Abstract

Maintaining cellular ploidy is critical for normal physiological processes, although gains in ploidy are frequently observed during development, tissue regeneration, and metabolism, and potentially contribute to aneuploidy, thereby promoting tumor evolution. Although numerous computational tools have been developed to estimate cellular ploidy from whole‐genome sequencing (WGS) data at bulk or single‐cell resolution, to the knowledge, no systematic comparison of their performance has been conducted. Here, a benchmarking study is presented of 11 methods for bulk WGS and 8 methods for single‐cell WGS data, utilizing both experimental and simulated datasets derived from diploid cells mixed with aneuploid or polyploid cells. For bulk WGS tools, their performance is evaluated in estimating tumor purity and ploidy, as well as the influence of preprocessing steps, somatic mutation callers, purity, sequencing platforms, and depths. It is found that PURPLE outperforms other methods when tumor purity exceeded 30%, regardless of sequencing coverage or platform. However, all existing tools performed poorly applied to euploid samples or long‐read sequencing data. For single‐cell WGS tools, their ploidy detection accuracy is assessed, and SeCNV is identified as the top‐performing method. These findings provide valuable guidance for future research on ploidy analysis and ongoing improvements in computational tools for single‐cell sequencing data.

## Graphical abstract

It comprehensively benchmarks computational tools for ploidy estimation from bulk and single‐cell whole‐genome sequencing data. The results highlight the strengths and limitations of each method under different conditions and identify PURPLE and SeCNV show top performance in bulk and single‐cell settings, respectively. This work offers practical guidance for tool selection in genomic research.

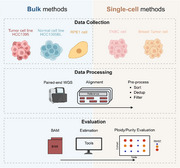

## 1 Introduction

Errors in DNA replication and/or abnormal chromosome segregation can result in changes in whole chromosome number, a phenomenon known as aneuploidy, or in doubling of the entire chromosome set, referred to as whole‐genome doubling (WGD).^[^ ^1^, ^2^ ^]^Developmentally programmed WGD can occur in several tissues,^[^ ^3^, ^4^, ^5^ ^]^ such as the liver,^[^ ^6^ ^]^ heart,^[^ ^7^ ^]^ bone marrow,^[^ ^8^, ^9^ ^]^ and breast.^[^ ^10^ ^]^ In contrast, unscheduled WGD has been observed in ≈30% of human cancers,^[^ ^11^ ^]^ promoting chromosomal instability (CIN) and facilitating the acquisition of additional aneuploidies.^[^ ^12^ ^]^ The prevalence of aneuploidy varies significantly across cancer types, ranging from ≈25% in thyroid cancer to ≈100% in glioblastoma.^[^ ^13^ ^]^ Importantly, aneuploidy has gradually recognized as an attractive biomarker, providing critical insights for clinical diagnosis, classification, prognosis, and therapy.^[^ ^13^ ^]^ In patients with invasive breast cancer, early‐stage endometrial cancer, early‐stage ovarian cancer, prostate cancer, and colorectal cancer, cellular ploidy serves as an independent prognostic indicator significantly associated with adverse outcomes.^[^ ^14^ ^]^ In colorectal cancer, higher rates of aneuploidy are observed in advanced tumors compared to early‐stage tumors, suggesting its potential utility as a staging marker.^[^ ^15^ ^]^ Consequently, accurate detection and quantification of cellular ploidy, as well as its proportion in cancer, are crucial for understanding the molecular mechanisms of cancer, developing precision therapies, and predicting patient responses to treatment.

Ploidy detection approaches have evolved from low‐resolution, low‐throughput techniques to high‐resolution, high‐throughput techniques. Traditional karyotyping allows direct observation of chromosomal morphology and numerical alterations under a microscope, but is limited by its low throughput, labor‐intensive procedures, and dependence on metaphase chromosome preparation, making it unsuitable for high‐throughput studies or complex samples.^[^ ^16^ ^]^ Fluorescence in situ hybridization (FISH) employs specific fluorescent probes to discover chromosomal abnormalities in non‐dividing cells. However, it is restricted to detecting known target regions and cannot provide a genome‐wide overview.^[^ ^17^, ^18^, ^19^ ^]^ Flow cytometry, another widely used method, measures cellular DNA content by employing dyes that bind stoichiometrically to DNA. This approach determines cell cycle distribution and ploidy status but requires appropriate ploidy controls and sufficient cells as input material.^[^ ^20^ ^]^ The advent of next‐generation sequencing (NGS) has ushered in a new era of low‐cost, high‐resolution aneuploidy detection. Whole‐genome sequencing (WGS) technology, in particular, facilitates high‐resolution detection of copy number variations (CNVs)^[^ ^21^, ^22^ ^]^ by read depth, which can then be further leveraged to infer ploidy alterations. Additionally, allele frequencies based on single nucleotide polymorphisms (SNPs), known as B‐allele frequency (BAF), enable absolute copy number estimation and tumor cell fraction assessment.^[^ ^23^ ^]^ Based on these features, an increasing number of computational tools have been developed to analyze WGS data for tumor cell purity, ploidy, and CNV detection, with widespread applications in cancer research. Although numerous benchmark studies have been conducted to evaluate the accuracy of these tools in detecting CNVs,^[^ ^24^ ^]^ their ability to estimate tumor cell purity and ploidy has often been overlooked.

The emergence of single‐cell sequencing technology has made it possible to measure ploidy at the individual cell level.^[^ ^25^ ^]^ Unlike traditional NGS technologies, which can only provide average signals from bulk populations, single‐cell DNA sequencing (scDNA‐seq) can reveal cell‐to‐cell ploidy heterogeneity and low‐frequency or rare subpopulations of cells with ploidy abnormalities, thereby offering a more detailed view of tumor genomic heterogeneity.^[^ ^26^ ^]^ Although several tools have been developed for single‐cell ploidy identification, it remains largely unclear how these methods compare to each other.

To facilitate the selection of ploidy estimation methods, we conducted a comprehensive benchmark of existing tools capable of ploidy estimation for both bulk and single‐cell sequencing data. Specifically, we evaluated 11 and 8 tools developed for bulk and single‐cell genome sequencing, respectively, and conducted a comprehensive analysis by integrating real tumor sample data with simulated datasets across various scenarios, focusing on their performance in tumor cell purity estimation, ploidy detection accuracy, and error bias direction. In summary, we highlight the potential limitations of these tools in resolving the complexity of tumor genomes, providing practical guidance for researchers to select the most appropriate tools based on specific use cases.

## 2 Results

### 2.1 Overview of Benchmarking Strategies

**Figure 1:**
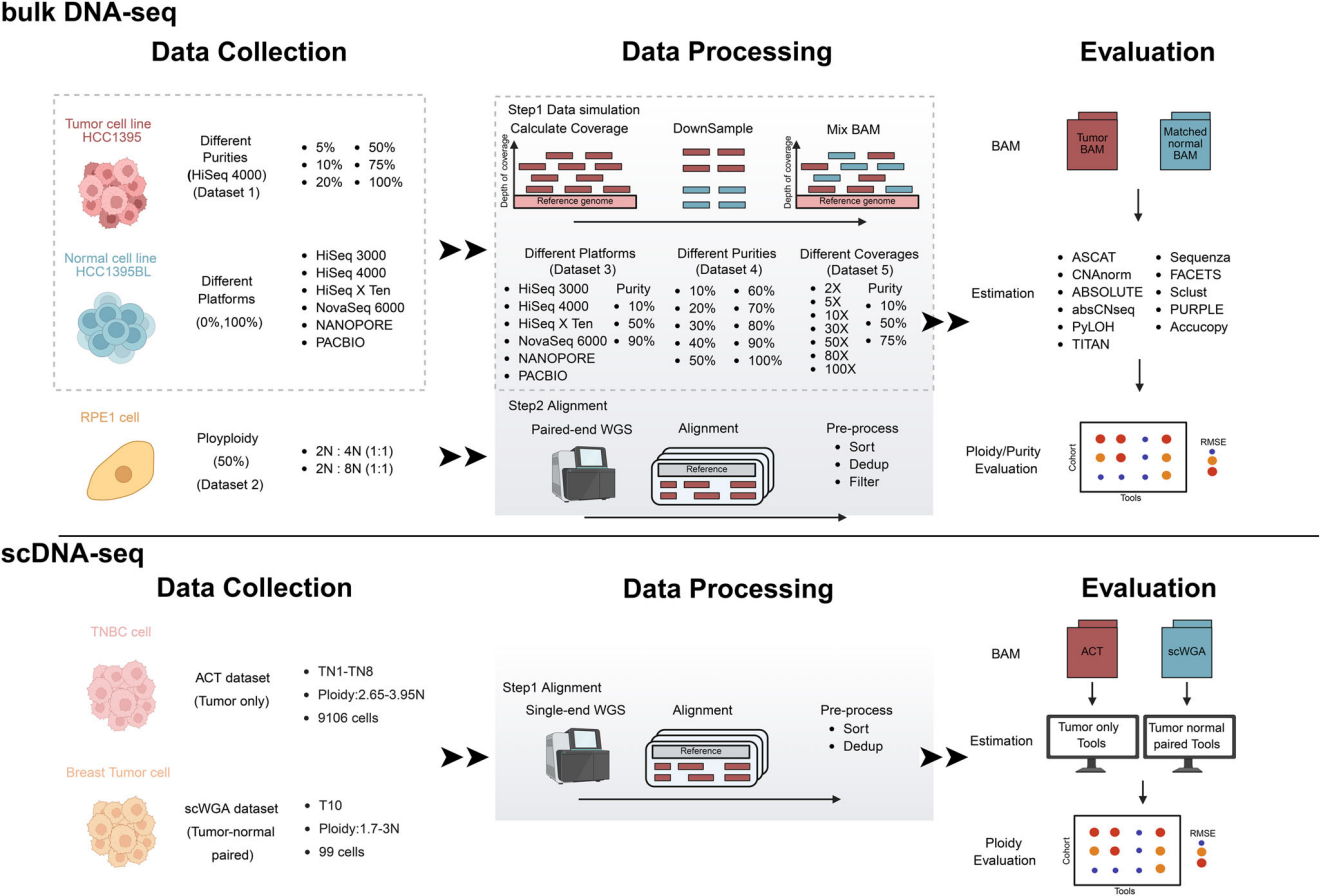
Schematic representation of the benchmarking workflow. This figure illustrates the workflow of this benchmarking study. The top panel shows the workflow for comparing bulk sequencing methods, and the bottom panel shows the workflow for scDNA‐seq approaches. HCC1395/HCC1395BL WGS data and scDNA‐seq datasets were collected from public repositories. Dataset 2 was in‐house generated data that designed for euploid studies. Simulated datasets were generated by calculating coverage (mosdepth) and down sampling (sambamba) from HCC1395/HCC1395BL data to create mixtures with different platforms (Dataset 3), purity (Dataset 4), and coverage (Dataset 5). In both workflows, software performance was evaluated across multiple scenarios. Estimation accuracy was quantified using the RMSE. For bulk sequencing methods, we evaluated purity and ploidy estimation accuracy, whereas for single‐cell sequencing methods, assessment focused solely on ploidy detection performance.

Given the differences in ploidy estimation between bulk and single‐cell whole genome sequencing data, we designed distinct strategies to evaluate the performance of different methods across various contexts (**Figure** **1**).

For bulk sequencing methods, we systematically assessed the accuracy of their purity and ploidy estimation across different platforms, sequencing coverages, and purity levels using both experimental and simulated data (Table S1, Supporting Information). We first included two experimentally generated datasets: 1) Dataset 1 consists of publicly available WGS data from the Sequencing Quality Control 2 (SEQC2) Consortium, using a polyploid breast cancer cell line HCC1395 (2.8*n*) and its matched diploid lymphocyte cell line, HCC1395BL, derived from the same individual.^[^ ^27^ ^]^ DNA from HCC1395 and HCC1395BL were pooled at various ratios (5%, 10%, 20%, 50% and 75%) and sequenced on the Illumina HiSeq 4000 platform;^[^ ^28^ ^]^ 2) Dataset 2 was generated in‐house to evaluate the resolution of euploid versus polyploid states, we induced WGD through mitotic slippage in RPE‐1 cells, which are hTERT‐immortalized retinal pigment epithelial cells, and then sorted the cells to generate WGS data by mixing diploid cells with an equal amount of tetraploid or octoploid cells (Figure S1, Supporting Information). To complement the experimental data and enable more controlled comparisons, we created three simulated datasets based on the SEQC2 data: 1) Dataset 3 was simulated for platform specific comparisons. We collected the WGS data of HCC1395 and HCC1395BL sequenced at six platforms (HiSeq 3000, HiSeq 4000, HiSeq X Ten, and NovaSeq 6000 for short‐read sequencing and PacBio and Oxford Nanopore for long‐read sequencing (LRS) from SEQC2, then simulated low‐, median‐, and high‐purity tumor samples for each platform based on the real data; 2) Dataset 4 was generated to evaluate performance across a finer gradient of tumor purity. In silico mixtures with tumor purity ranging from 10% to 90% in 10% steps were created using HCC1395 and HCC1395BL WGS data; 3) Dataset 5 was simulated to assess the impact of sequencing depth. We subsampled the Dataset 1 mixtures to generated data at different coverages (2X, 5X, 10X, 30X, 50X, 80X, and 100X) with low, medium, and high tumor purity. The accuracy of purity estimation was mainly measured by the root mean squared error (RMSE) and the Pearson correlation coefficient (PCC), while the discrepancy between estimated and true ploidy was quantified by RMSE.

As single‐cell sequencing methods can directly determine the ploidy of individual cells, inherently avoiding the need for purity assessment, we evaluated their ability to detect cellular ploidy using two published datasets that contain orthogonal information on ploidy states. One dataset comprises 8 cell clusters profiled using the acoustic cell tagmentation (ACT) protocol,^[^ ^20^ ^]^ with ploidy ranging from 2.65N to 3.95N. Due to a lack of diploid normal cells, this dataset could not be applied to the “Tumor‐normal paired” tools such as SCOPE and CNVeil. We therefore also collected a second dataset that sequenced 99 cells (exclude a cell with no mapped reads) with ploidy ranging from 1.7N to 3.3N profiled by single‐cell whole genome amplification (scWGA) protocol^[^ ^29^ ^]^ for both “Tumor‐only” and “Tumor‐normal paired” methods. The ploidy estimation accuracy was measured by RMSE and the proportion of outliers whose absolute difference between the inferred and true ploidy exceeded 0.2.

### 2.2 Assessment of Bulk Sequencing Methods in Tumor Ploidy and Purity Estimation

A total of 11 computational methods were considered in this study for bulk WGS. Ten of them utilize information from both read depth and BAF, including ASCAT,^[^ ^30^ ^]^ ABSOLUTE,^[^ ^31^ ^]^ absCNAseq,^[^ ^32^ ^]^ PyLOH,^[^ ^33^ ^]^ TITAN,^[^ ^34^ ^]^ Sequenza,^[^ ^35^ ^]^ FACETS,^[^ ^36^ ^]^ Sclust,^[^ ^37^ ^]^ PURPLE^[^ ^38^ ^]^ and Accucopy,^[^ ^39^ ^]^ while the other algorithm, CNAnorm,^[^ ^40^ ^]^ relies only on read depth (Table S2, Supporting Information).

**Figure 2:**
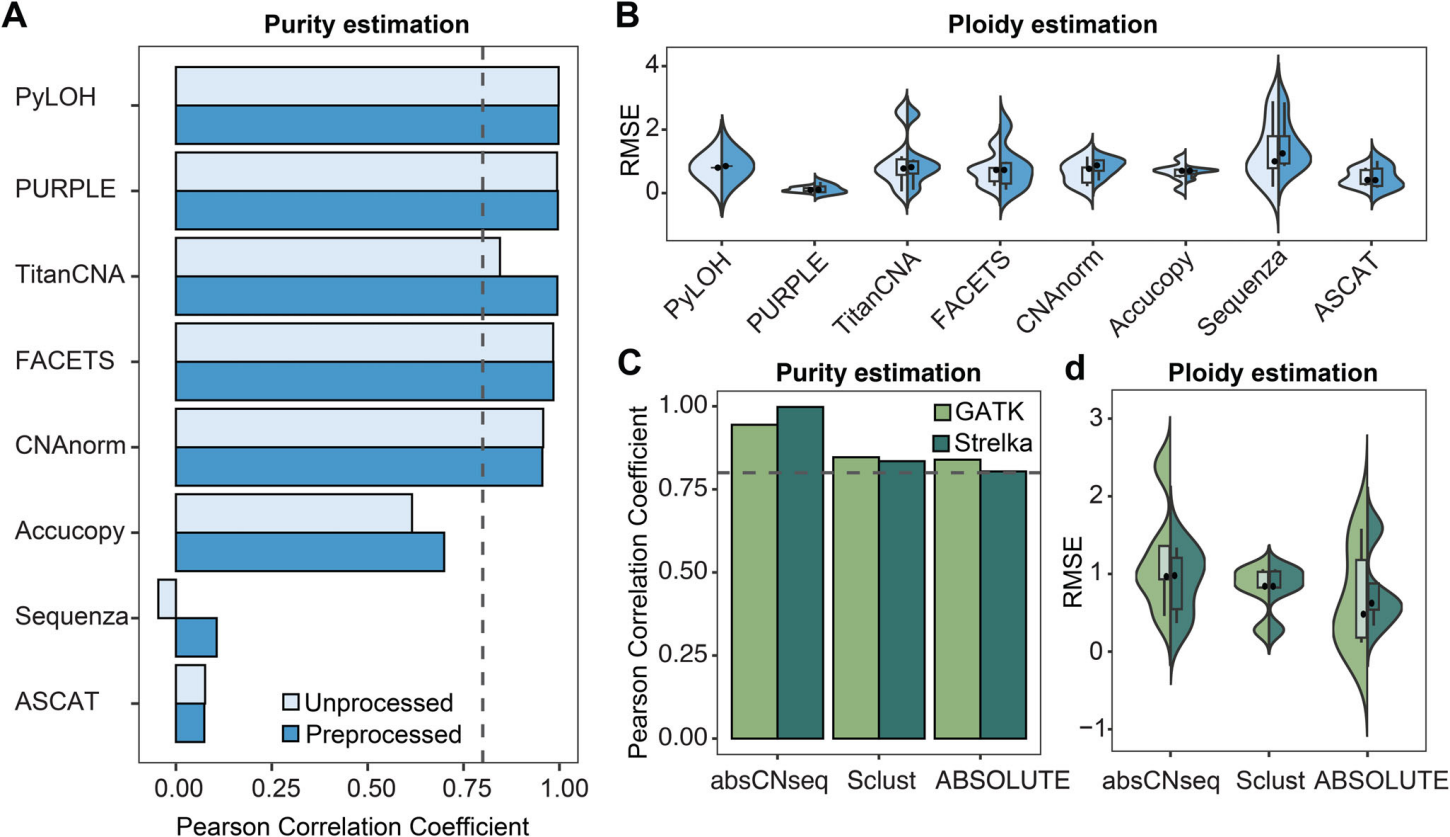
Impact of alignment pre‐processing and variant calling strategies on tumor purity and ploidy estimation. A,B) Impact of alignment pre‐processing on tumor purity (A) and ploidy (B) estimation for 8 tools that do not require SNP file as input. C,D) Impact of variant calling strategies on tumor purity (C) and ploidy (D) estimation for 3 tools that require SNP file input. Pearson correlation coefficients (r) were used to assess the correlation between estimated and true tumor purity for each tool (r > 0.8 indicates reliable estimation, gray dashed line). Ploidy estimation accuracy quantified by RMSE (lower values indicate higher precision).

To standardize our analysis pipeline prior to ploidy estimation, we first evaluated the impact of alignment pre‐processing and variant calling strategy selection. Although pre‐processing steps such as PCR duplicate removal and low‐quality alignment filtering are standard practice in WGS data analysis, they were not explicitly described in the user manuals of the evaluated tools. By analyzing purity and ploidy of Dataset 1, we found that alignment preprocessing slightly improved the accuracy of tumor purity estimation for TitanCNA, Accucopy, and Sequenza, without significantly affecting ploidy estimation (**Figure** **2**A,B). Furthermore, although most methods incorporate built‐in modules for variant identification, ABSOLUTE, absCNAseq, and Sclust require users to provide mutation files as input. The usage of BAF clearly improved their estimation performance (Figure S2, Supporting Information), but it remains unclear whether different mutation calling workflows would affect the results. Comparing the two widely used variant calling strategies, GATK^[^ ^41^ ^]^ and Strelka,^[^ ^42^ ^]^ we observed no substantial differences in either tumor purity or ploidy estimation (Figure 2C,D). However, Strelka completed variant calling more than 17‐fold faster than GATK.^[^ ^43^ ^]^ Based on these findings, we standardized the alignment processing steps and adopted Strelka for all subsequent analyses.

**Figure 3:**
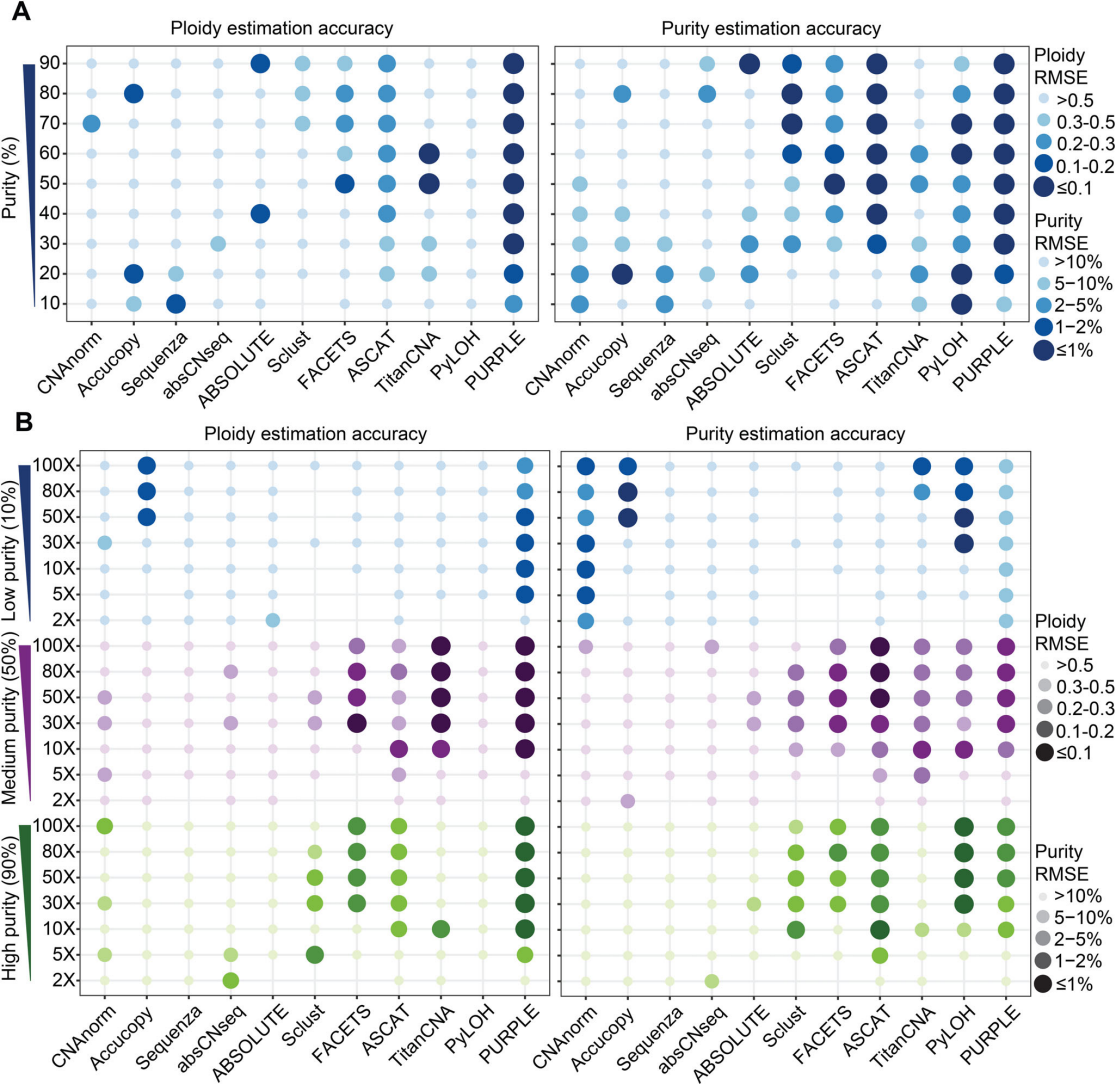
Impact of tumor purity and sequencing coverage on tumor purity and ploidy estimation. A) Impact of tumor purity on ploidy (left) and purity (right) estimation accuracy across 9 purity gradients (10–90%, step size 10%) in 3. Each gradient includes three replicates. B) Impact of sequencing coverage on ploidy (left) and purity (right) estimation accuracy under low (10%)‐, medium (50%)‐, and high (75%)‐purity conditions in Dataset 5. Coverage levels include 2X, 5X, 10X, 30X, 50X, 80X, and 100X, with three replicates per condition. Dot size and color intensity represent estimation accuracy; larger and darker dots indicate higher accuracy. Ploidy estimation is considered accurate if RMSE ≤ 0.2, and purity estimation is considered accurate if RMSE ≤ 2%.

We next systematically benchmarked the influence of tumor purity using Dataset 4, which spans a comprehensive gradient. Ploidy estimation was considered reliable when the RMSE was < 0.2, while purity estimation was considered accurate if RMSE values fell below 2%. Notably, performance varied substantially across purity ranges among different methods. At low purity levels (≤20%), most tools exhibited limited accuracy in both purity and ploidy estimation, consistent with previous findings in copy number analysis.^[^ ^39^ ^]^ Accucopy and PURPLE provided accurate purity and ploidy estimates at 20% purity, whereas PyLOH achieved reliable purity estimates at both 10% and 20%. However, PyLOH consistently reported a fixed ploidy value of 2, irrespective of the true ploidy. In intermediate‐to‐high purity samples (≥30%), ASCAT and PURPLE outperformed the other methods. Specifically, ASCAT delivered robust purity and ploidy estimates at purities of ≥50%, while PURPLE maintained high accuracy across the entire intermediate‐to‐high purity range (**Figure** **3**A). Specifically, PURPLE maintained high accuracy across intermediate‐to‐high purity ranges, whereas ASCAT achieved robust estimates within this range despite exhibiting a systematic ploidy overestimation bias of approximately +0.24 compared to true ploidy (Figure S3A, Supporting Information).

The minimum sequencing coverage required for tools to achieve robust estimation directly impacts their practical cost‐effectiveness. To assess tool performance across different sequencing coverages, we analyzed Dataset 5, which includes low‐, medium‐, and high‐purity samples with coverages ranging from 2× to 100×. In low purity samples, Accucopy showed superior detection capabilities compared to other tools aligning with its specialized design for low‐purity contexts (Figure 3b).^[^ ^39^ ^]^ Notably, Accucopy achieved accurate purity and ploidy estimation only at coverage levels greater than 50×, The inconsistent performance of Accucopy that poor accuracy in Dataset 4 (10% purity) versus improved results in Dataset 5 (10% purity), likely stems from its limited stability across replicates (Figure S4A,B, Supporting Information). In contrast, PURPLE demonstrated robust accuracy for medium‐ and high‐purity samples, achieving stable estimates even at 10× coverage, with performance plateauing beyond this threshold (Figure 3B). Meanwhile, for low‐purity samples, it exhibited a systematic bias toward overestimating both tumor ploidy and purity (Figure S3B, Supporting Information).

**Figure 4:**
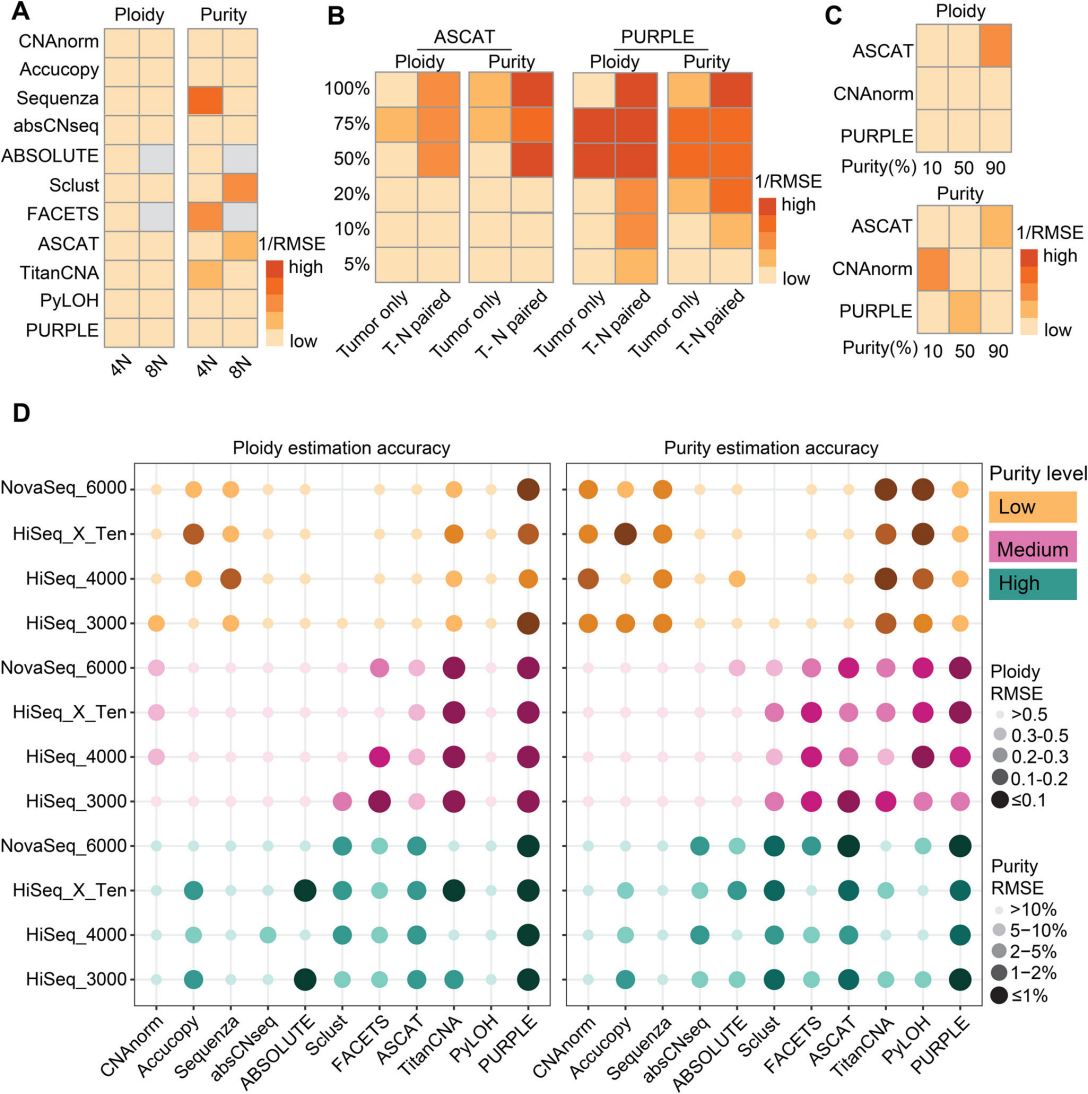
Accuracy of tumor purity and ploidy estimation across special scenarios and platforms. A) Accuracy of ploidy (left) and purity (right) estimation by each tool using Dataset 2, which includes 4N and 8N samples with three replicates each. B) Accuracy of ploidy (left) and purity (right) estimation by ASCAT and PURPLE in tumor‐only mode and tumor‐normal paired mode. C) Accuracy of ploidy (left) and purity (right) estimation using long‐read data (PacBio, Dataset 3) under low‐, medium‐, and high‐purity gradients, with three replicates per condition. D) Impact of four short‐read sequencing platforms (Dataset 3) on the accuracy of ploidy (left) and purity (right) estimation under low‐, medium‐, and high‐purity gradients, with three replicates per condition. Dot size and color intensity represent estimation accuracy; larger and darker dots indicate higher accuracy. Ploidy estimation is considered accurate if RMSE ≤ 0.2, and purity estimation is considered accurate if RMSE ≤ 2%. Heatmaps display accuracy as 1/RMSE. Five accuracy intervals are defined based on the RMSE value ranges derived from dot plot segmentation (for both ploidy and purity). Darker colors indicate higher accuracy. The top two darkest color blocks represent the accurate estimations.

Next, we investigated whether these tools could distinguish euploid cells, such as WGD events occurring during early tumorigenesis and tissue development. Analysis of Dataset 2 revealed that most approaches failed to accurately determine ploidy in diploid samples containing mixtures of tetraploid or octoploid cells, with RMSE values substantially above 0.2 (corresponding to 1/RMSE<5) (**Figure** **4**A). This poor performance is likely due to the heavy reliance of most tools on BAF for ploidy inference, which is highly limited in WGD cells and thus confounds accurate ploidy estimation. Furthermore, although most bulk sequencing methods operate in a “Tumor‐normal paired” (T‐N paired) mode, ASCAT and PURPLE additionally provide “Tumor‐only” mode, expanding their applicability. Both ASCAT and PURPLE exhibited reduced ploidy estimation accuracy in tumor‐only mode compared to their T‐N paired mode. However, PURPLE still achieved reliable purity and ploidy estimates at 50% and 75% purity levels, albeit with modestly lower precision in tumor‐only mode (Figure 4B). Therefore, for samples lacking matched diploid normal controls, we recommend PURPLE for purity and ploidy estimation.

We further evaluated the effects of different sequencing platforms, including both short‐read and LRS technologies. Most tools exhibited cross‐platform consistency across short‐read platforms, although some exceptions were noted., TitanCNA, for instance, demonstrated higher accuracy for data generated from HiSeq 3000 and HiSeq X Ten with 90% purity but performed poorly on data from NovaSeq 6000 and HiSeq 4000, consistent with results from Dataset 4 derived from HiSeq 4000 (Figure 2A). At this purity level, despite platform‐specific accuracy variations, TitanCNA exhibited a consistent directional bias across all platforms, systematically overestimating ploidy and underestimating purity (Figure S3C, Supporting Information). For LRS data, none of the tools successfully analyzed Nanopore sequencing data. Among the three tools supporting PacBio data, only ASCAT explicitly advertises long‐read compatibility. However, none achieved accurate ploidy and purity estimation on long‐read platforms, suggesting unresolved technical challenges in adapting existing algorithms to these modalities (Figure 4C).

### 2.3 Assessment of Single‐Cell Sequencing Methods in Ploidy Estimation

We benchmarked eight ploidy estimation tools for single‐cell sequencing, including six methods that provide both tumor‐only and tumor‐normal (T‐N) paired modes: HMMcopy,^[^ ^44^ ^]^ Ginkgo,^[^ ^45^ ^]^ AneuFinder,^[^ ^46^ ^]^ SeCNV,^[^ ^47^ ^]^ rcCAE^[^ ^48^ ^]^ and scAbsolute,^[^ ^49^ ^]^ and two methods that support only the T‐N paired mode, SCOPE^[^ ^50^ ^]^ and CNVeil (https://github.com/maiziezhoulab/CNVeil) (Table S3, Supporting Information).

**Figure 5:**
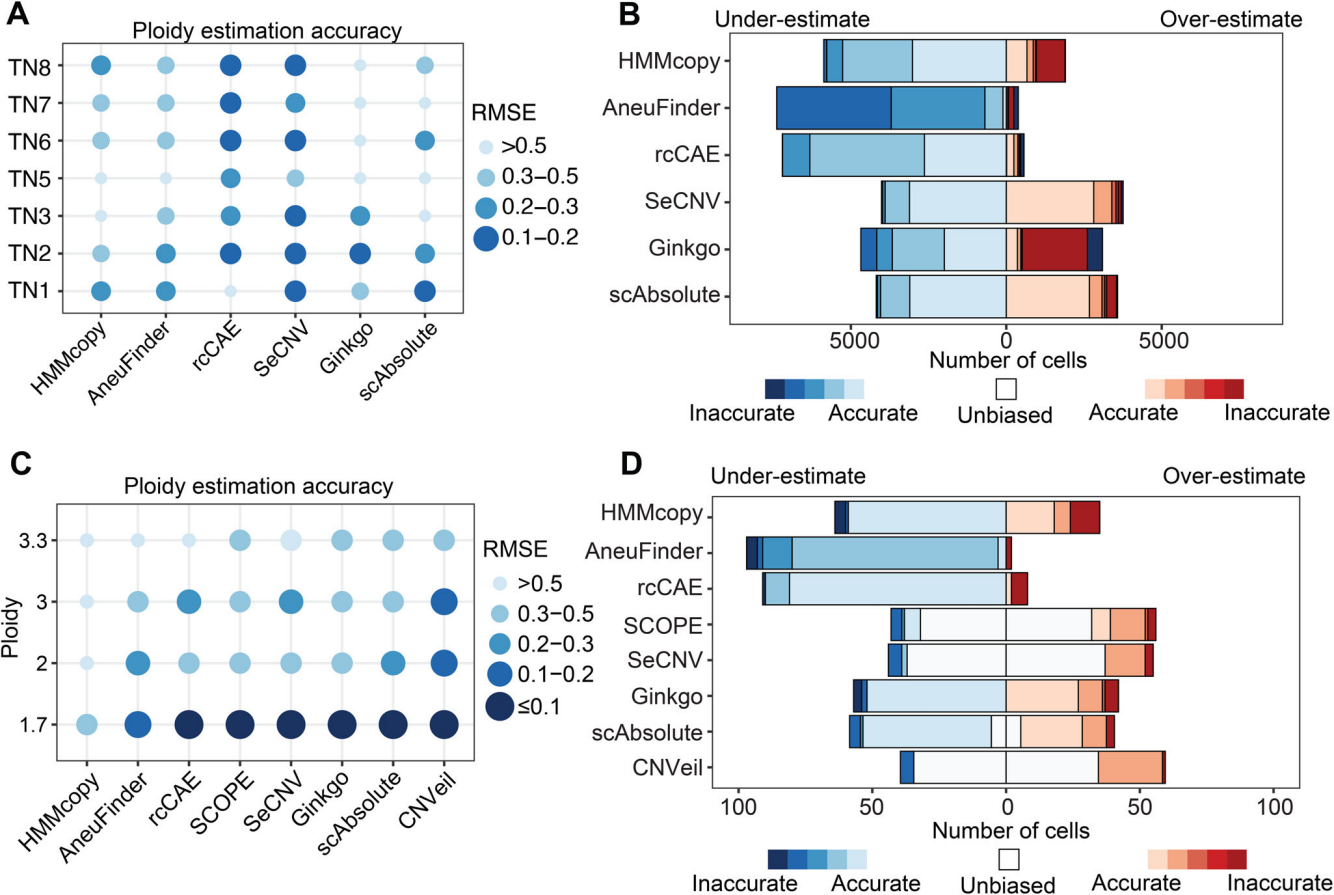
Accuracy and bias in tumor ploidy estimation from scDNA‐seq datasets. A,B) Accuracy of ploidy estimation (A) and bias between the measured and true ploidy values (B) by different tools in the ACT dataset. C,D) Accuracy of ploidy estimation (C) and bias between the measured and true ploidy values (D) by different tools in the scWGA dataset. Dot size and color intensity represent estimation accuracy; larger and darker dots indicate higher accuracy. Ploidy estimation is considered accurate if RMSE ≤ 0.2, and purity estimation is considered accurate if RMSE ≤ 2%. For the stacked bar plot, blue indicates under‐estimation and red indicates over‐estimation, with deeper colors representing greater deviation. White denotes no difference between estimated and true values.

Using the ACT dataset, we first examine the performance of these tools in “Tumor‐only” condition. Among them, SeCNV outperformed others, achieving accurate ploidy estimates across all cell clusters except TN4 and TN5 (**Figure** **5**A), with the lowest mean outlier proportion of 10.8% (Table S4, Supporting Information). For TN4 and TN5, especially TN4, however, all tools exhibited elevated outlier rates (Table S4, Supporting Information), suggesting potential intrinsic flaws in these datasets. The poor performance of TN4 has also been observed before and attributed to an “unidentifiability” issue,^[^ ^49^ ^]^ thus we excluded TN4 in the following analysis to avoid confusion. Additionally, rcCAE also demonstrated robust ploidy estimation for most ACT clusters (Figure 5A). Most approaches tend to underestimate cellular ploidy, especially AneuFinder (Figure 5B).

Next, we used the scWGA dataset to evaluate these tools’ performance under T‐N paired condition, CNVeil, SCOPE, SeCNV, and scAbsolute all showed strong performance (Figure 5C), with mean ploidy estimation errors below 0.1 (Table S4, Supporting Information). CNVeil achieved the highest accuracy, but its superiority may be attributed to prior optimization and testing on this specific scWGA dataset, limiting generalizability. Furthermore, unlike “Tumor‐only” tools such as SeCNV, CNVeil, and SCOPE require diploid normal cells as additional inputs, restricting their applicability. And in this dataset, both Aneufinder and rcCAE exhibited severe global underestimation of tumor ploidy (Figure 5D).

Altogether, based on a comprehensive assessment, SeCNV is recommended for single‐cell ploidy estimation. Although SeCNV's CPU time is not the shortest among the tested tools (Figure S4D, Supporting Information), processing 100 cells can still be completed within 1 h using a multi‐core computing system.

### 2.4 Stability and Computational Performance Analyses

A major challenge in tumor purity and ploidy estimation lies in their intrinsic coupling: identical genomic profiles can be explained by multiple combinations of purity and ploidy, leading to non‐unique solutions. This ambiguity is further exacerbated in cases of polyploidy induced by WGD, where BAF signals are often uninformative. As a result, estimation stability across replicates and repeated runs has become a critical performance metric for evaluating existing tools. Our results show that CNAnorm exhibits the poorest stability across both replicates and repeated runs. Most tools demonstrate robust stability within their optimal purity ranges (Figure S4A,B, Supporting Information). For instance, PURPLE maintains high accuracy and stability across all purity levels, whereas ASCAT performs reliably in samples with intermediate‐to‐high purity. In contrast, ABSOLUTE exhibits pronounced instability, possibly due to its tendency to output all mathematically equivalent solutions without prioritizing biologically plausible purity‐ploidy combinations.

We evaluated the computational efficiency of bulk and single cell tools focusing on two metrics: CPU time and Random Access Memory (RAM) usage. For bulk methods, efficiency was assessed on WGS data with 100× coverage from Dataset 5, while single‐cell tools were evaluated on the scWGA data containing 99 cells. Results showed that ASCAT, PURPLE, CNAnorm, and TitanCNA demonstrated superior computational performance, with shorter CPU times and lower RAM usage (Figure S4C, Supporting Information). Specifically, ASCAT achieved the shortest CPU time, whereas TitanCNA consumed the lowest RAM. For single‐cell methods, Aneufinder, rcCAE, and Ginkgo outperformed others in computational efficiency. In contrast, SeCNV, the top performer in ploidy estimation, required significantly higher CPU time and RAM usage. Nevertheless, processing 99 cells with SeCNV could still be completed within an hour using a multi‐core computing system (Figure S4D, Supporting Information).

## 3 Discussion

**Figure 6:**
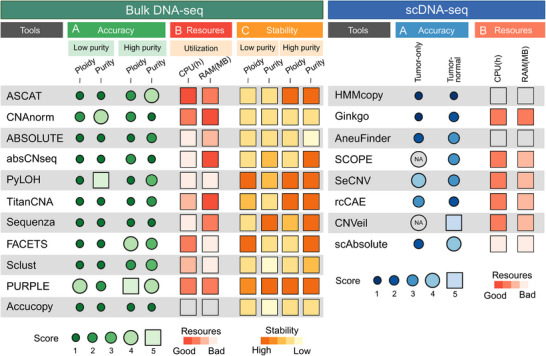
Overview of benchmarking results across all datasets, including accuracy, usability, and scalability. A) Summary of the accuracy of purity and ploidy estimation by each tool at 30× coverage under both low‐ and high‐purity conditions. For ploidy estimation, scores from 1 to 5 correspond to RMSE >0.5, 0.3–0.5, 0.2–0.3, 0.1–0.2, and ≤0.1, respectively. For purity estimation, scores from 1 to 5 correspond to RMSE >10%, 5–10%, 2–5%, 1–2%, and ≤1%, respectively. B) Resource usage of different tools across all datasets, with darker colors indicating lower usage. C) Stability of results in bulk datasets, measured by the coefficient of variation across three replicates, with darker colors indicating higher stability.

Accurate identification of cellular ploidy is crucial for understanding genomic instability, tumor evolution, and therapeutic response in cancer research. In this study, we conducted a systematic benchmarking of 11 bulk and 8 single‐cell sequencing tools, assessing their performance in tumor purity and ploidy detection across diverse biological scenarios to identify the most robust analytical methods. Among the tools we examined, PURPLE demonstrated the highest accuracy for bulk sequencing, with top‐tier stability and resource efficiency, while SeCNV emerged as the most robust single‐cell sequencing method (**Figure** **6**). However, the performance varied contextually: PURPLE showed reduced accuracy in low‐purity samples, particularly for purity estimation, while PyLOH achieves better purity estimation accuracy at low‐purity conditions but with poor ploidy estimation. Despite its widespread use, RMSE is sensitive to outliers and technical variability. In our analysis, tools such as Accucopy, CNAnorm, absCN‐seq, and ABSOLUTE showed considerable fluctuations across replicate runs of the same experimental sample, likely due to factors such as batch effects or sensitivity to minor input perturbations. This inconsistency inflates RMSE and may obscure genuine improvements driven by higher tumor purity or sequencing coverage. While ABSOLUTE showed modest improvement in simulated purity gradients, other tools remained unstable, highlighting limitations in their robustness.

Emerging LRS technologies now offer a powerful alternative to NGS, enabling more accurate genome assembly and comprehensive detection of complex structural variants as well as DNA modifications.^[^ ^51^ ^]^ The two major strategies of LRS are the single‐molecule real‐time sequencing (or PacBio sequencing) offered by Pacific Bioscience and nanopore sequencing from Oxford Nanopore Technologies (ONT), greatly contributing to the recent complete human reference genome T2T‐CHM13 by Telomere‐to‐Telomere Consortium.^[^ ^52^ ^]^ Despite LRS having been rapidly adopted and widely implemented in current research, our benchmarking reveals existing computational methods for ploidy estimation remain either incompatible with or demonstrate suboptimal performance on this platform, emphasizing an urgent need to develop new computational methods specifically designed for long‐read sequencing to enable accurate ploidy and tumor purity detection.

Ploidy alterations occur not only in cancers as aneuploidy, but also participate in normal developmental and differentiation processes. Taking hepatocyte as an example, polyploidization takes place during liver homeostasis, regeneration, and damage response. While most algorithms of ploidy estimation designed for cancer research based on BAF, our benchmarking result reveals their poor performance in identifying euploidy in normal cells that lack sufficient mutations. Thus, our findings highlight the need for developing mutation‐independent algorithms as an attractive future direction in ploidy analysis.

Together, this study provided a comprehensive comparison of available ploidy estimation methods of bulk and single‐cell sequencing data. Our results are a useful resource for guiding biologists who want to analyze ploidy abnormalities or computational scientists for further algorithm development.

## 4 Experimental Section

### Cell Culture and Treatment

hTERT RPE‐1 cells were grown in Dulbecco's modified medium (DMEM) F12 (Gibco, cat. no. 11320033) containing 10% fetal bovine serum (FBS) (Gibco, cat. no. A5256701), 100 U mL^−1^ Penicillin‐Streptomycin (Gibco, cat. no. 15140122), 10 µg mL^−1^ hygromycin B (Gibco, cat. no. 10687010). hTERT RPE‐1 cells were maintained in an incubator at 37 °C with 5% CO2 and tested negative for mycoplasma. To induce tetraploidy or octoploidy, hTERT RPE‐1 cells were incubated with 50 µm monastrol (Selleckchem, cat. no. S8439) and 1 µm MPI‐0479605 (Selleckchem, cat. no. S7488) for 24 h.

### Cell Sorting and Whole Genome Sequencing

For cell cycle profiling, cells pulsed with 20 µm EdU for 1 h were harvested and stained using Click‐iT EdU Alexa Fluor 647 Flow Cytometry Assay Kit (Invitrogen, cat. no. C10424) according to the manufacturer's instructions. For DNA content analysis, cells were measured using 1 µg mL^−1^ DAPI (Sigma‐Aldrich, cat. no. MBD0015). Finally, cells were resuspended in PBS and isolated by sorting for 2N, 4N, 8N DNA content using a BD FCASAria Fusion flow cytometer. Data were analysed using FlowJo v.10 software.

Sorted cells were collected, and their genomic DNA (gDNA) was extracted using the Quick‐DNA FFPE MiniPrep (Zymo cat. no. D3067). 2N/4N, 2N/8N gDNA dilutions was made in 1:1 ratio. 10 ng of 2N or mixed gDNA was used for the WGS libraries in triplicate. WGS libraries were prepared using the VAHTS Universal Plus DNA Library Prep Kit (Vazyme cat. no. ND617) according to the manufacturer's protocol and sequenced on a DNBSEQ‐T7 instrument (MGI Tech) at 2 × 150 bases read length.

### Data Simulation and Processing

A summary of the datasets used in this study is presented in Table S1 (Supporting Information). For the bulk section, short reads generated by Illumina were aligned to hg38 using BWA mem (v0.7.18)^[^ ^53^ ^]^ with –M option, while long reads were aligned to hg38 using Minimap2 (v2.26)^[^ ^54^ ^]^ with ‐ax map‐pb option for PacBio and ‐ax map‐ont option for Nanopore. For single‐cell section, all sequencing data were aligned to hg19 using BWA mem with –M option. Dataset 1 was 150 bp paired‐end reads with ≈140× coverage sequencing by Illumina HiSeq4000. Based on this dataset, nine distinct impure tumor samples (10%, 20%, 30%, 40%, 50%, 60%, 70%, 80%, 90%), designated as Dataset 4, were generated by proportionally mixing sequencing data from the pure tumor sample and its matched normal sample using an in‐house developed script. Additionally, down‐sampling was performed on the 10%, 50%, and 75% purity samples in this dataset to simulate samples with varying coverages (2X, 5X, 10X, 30X, 50X, 80X, 100X) under these purity levels, forming Dataset 5. Dataset 3, representing cross‐platform simulated data, was created by mixing sequencing data from platform‐specific pure tumor samples and their matched normal samples at ratios of 10%, 50%, and 90%. All mixing and down‐sampling operations were performed using sambamba (v1.0.1),^[^ ^55^ ^]^ while coverage calculations for all samples were rapidly conducted via mosdepth (v0.3.8).^[^ ^56^ ^]^ During preprocessing, PCR duplicate removal was performed using Picard (v3.1.1) (https://broadinstitute.github.io/picard/), while low‐quality alignment filtering was conducted via SAMTools view (v1.9)^[^ ^57^ ^]^ with the ‐q 37 option.

### SNP Calling and Segmentation Files Generation

The SNP files used in the analysis were generated through Strelka (v2.9.2)^[^ ^58^ ^]^ and GATK (v4.1.7.0)^[^ ^41^ ^]^ pipelines. For Strelka, SNPs were called using default parameters. For GATK, the standard workflow was followed: base quality score recalibration was first performed with BaseRecalibrator and ApplyBQSR, followed by SNP calling and filtering using Mutect2 and FilterMutectCalls. The resulting SNP files in Variant Call Format (VCF) were converted to Mutation Annotation Format (MAF) via vcf2maf (v1.6) (https://zenodo.org/records/1185418) to serve as input for the ABSOLUTE. Additionally, both PyLOH and ABSOLUTE require segmentation files as input. These files were generated following the GATK standard workflow, which sequentially applies the following commands: CollectReadCounts, CreateReadCountPanelOfNormals, DenoiseReadCounts, and ModelSegments.

### Performance Evaluation of Each Tool

For ploidy and purity estimation, all tools were executed strictly following their official manuals, with default parameters applied unless specified. For ABSOLUTE and absCNseq, the maximum ploidy range was set to 4, while other parameters remained default. For PyLOH, though the segments file was optional, it was included as additional input based on the developer's recommendation. Estimation accuracy was assessed using RMSE to measure deviations from ground truth and Mean Error (ME) to evaluate systematic bias. For this study, estimation accuracy was categorized into five tiers based on RMSE thresholds: Very Accurate (score 5), Accurate (score 4), Fairly Accurate (score 3), Fairly Inaccurate (score 2), and Inaccurate (score 1). Specifically, ploidy estimates were classified as Very Accurate (RMSE < 0.1), Accurate (0.1–0.2), Fairly Accurate (0.2–0.3), Fairly Inaccurate (0.4–0.5), and Inaccurate (≥0.5), while purity estimates used thresholds of Very Accurate (RMSE < 0.01), Accurate (0.01–0.02), Fairly Accurate (0.02–0.05), Fairly Inaccurate (0.05–0.1), and Inaccurate (≥0.1). These tiers directly correspond to the scores illustrated in Figure 6. Note that “purity” strictly refers to the proportion of tumor cells in a mixed sample; however, all values shown in the figure represent DNA mixing ratios. For simplicity, the term “purity” was retained in this context. In practice, purity accuracy was estimated by converting DNA mixing ratios into the proportion of tumor cells in the mixed sample.

To evaluate computational performance, the GNU time program was used to record CPU time and RAM usage during analysis of 100× coverage WGS data for bulk and the scWGA data for single cell. The command /usr/bin/time ‐f “TIME_STATS∖t%e∖t%U∖t%S∖t%M” was employed to output: Wallclock time (%e), User‐space time (%U), Kernel‐space time (%S), and Peak memory consumption (%M). CPU time was calculated as the sum of user‐space and kernel‐space times, while RAM usage corresponded directly to peak memory consumption.

### Data Visualization

In R(version 4.3.1), the RMSE and ME were calculated to evaluate the distances between the measured and true values. To explore the standard preprocessing pipeline, the Pearson correlation coefficient was computed using the cor function. All dot plots and histograms were generated using the ggplot2 package (v3.5.1), while split‐violin plots were created with the introdataviz package (v 0.0.0.9003). Heatmaps were constructed using the pheatmap package (v 1.0.12).

## Conflict of Interest

The authors declare no conflict of interest.

## Author Contributions

Y.S. and Z.M. contributed equally to this work. Y.S., Z.M., and W.W. developed the methodology. S.W. and W.W. conceptualized and supervised the study. S.W. carried out experiments. Q.Z., Q.Y., J.G., L.Z., and Y.L. provided extra technical assistance. Y.S., Z.M., and W.W. wrote the manuscript. All authors read and approved the final manuscript.

## Supporting information



Supporting Information

## Data Availability

Newly generated whole genome sequencing data have been deposited in the Sequence Read Archive (SRA) database under the accession number PRJNA1256151.
