# Supplementary material for: Benchmarking Ploidy Estimation Methods for Bulk and Single‐Cell Whole Genome Sequencing
Source: Adv Sci (Weinh). 2025 Sep 8;12(45):e07839. doi: 10.1002/advs.202507839 (PMC12677682; doi:10.1002/advs.202507839)
Supplement: Supplementary file 1 — Supporting Information [file ADVS-12-e07839-s001.docx]

Supporting Information

Benchmarking ploidy estimation methods for bulk and single-cell whole genome sequencing

Yawei Song, Zilv Mei, Qijie Zheng, Qingqing Yuan, Yu Liang, Jiaqi Gao, Lang Zhou, Shuheng Wu*, Wei Wu*


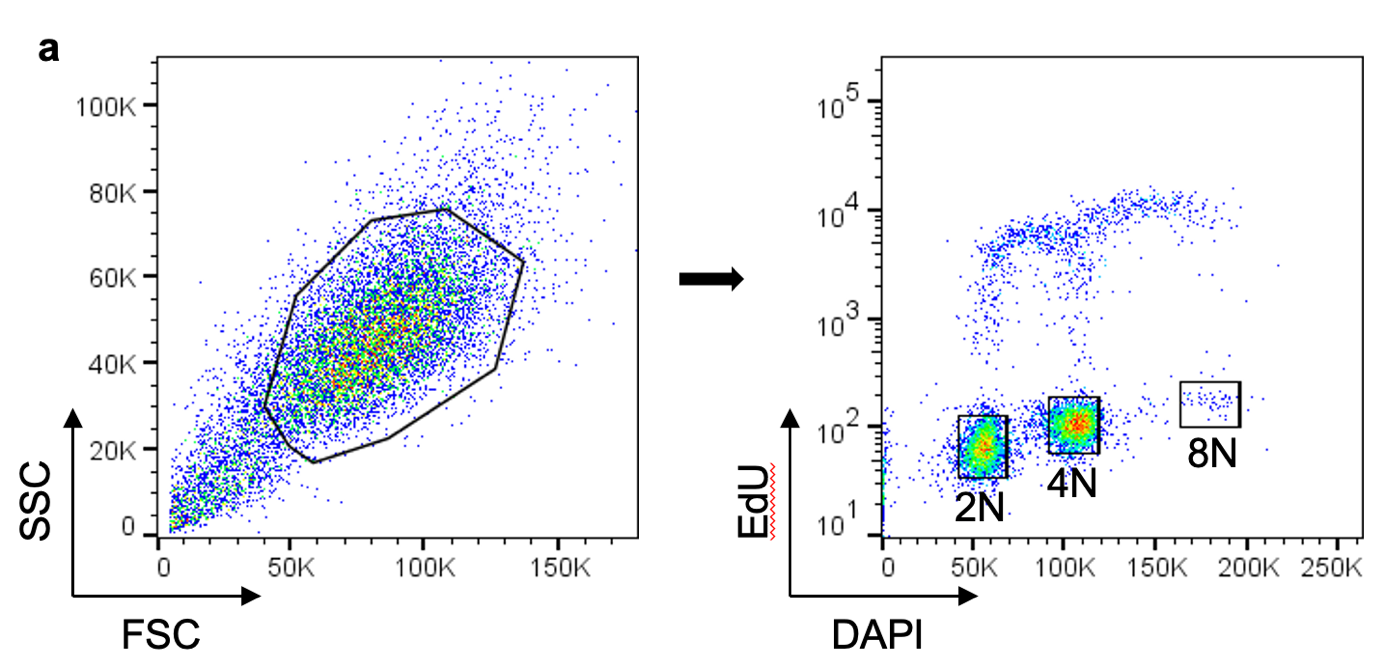


**Figure S1. FACS analysis of RPE1 cells incorporating EdU after induction of whole genome duplication.**

(A) FACS analysis of RPE1 cells incorporating EdU after induction of whole genome duplication by monastrol and MPI-0479605. gDNA were extracted from sorted 2N (diploid), 4N (tetraploid) and 8N (octoploid) cells for WGS library preparation.

**
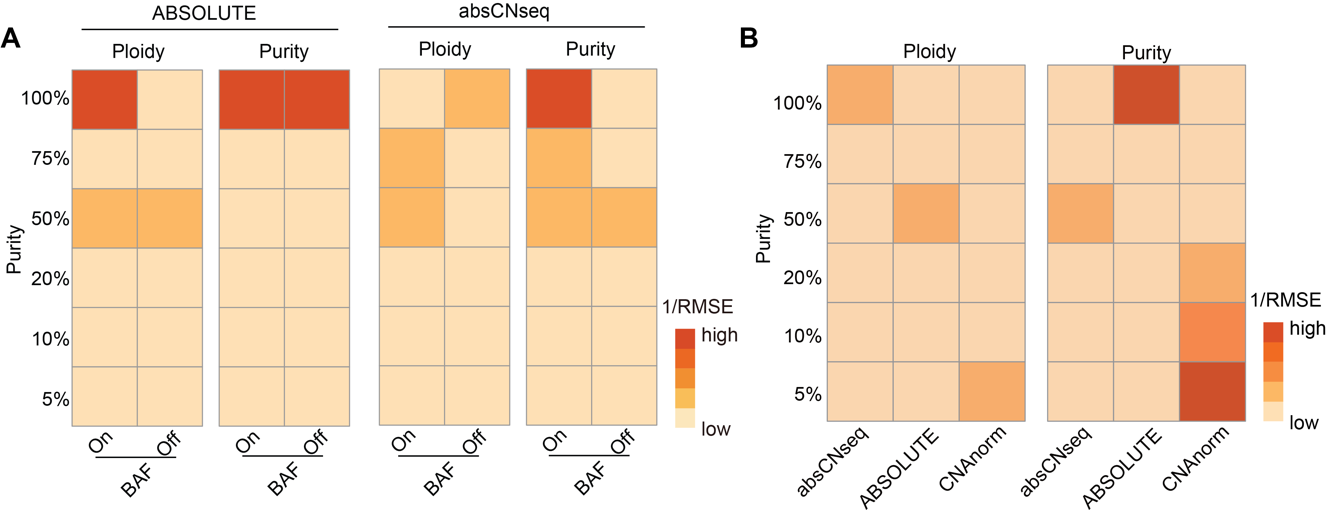
**

**Figure S2. Accuracy of tumor purity and ploidy estimation by different tools with and without BAF information.**

(A) Comparison of the accuracy of purity and ploidy estimation for Dataset 1 by ABSOLUTE (left) and absCN-seq (right), with or without the inclusion of additional somatic SNP files ("On" vs. "Off"). (B) Comparison of the accuracy of purity and ploidy estimation for Dataset 1 among ABSOLUTE and absCN-seq (both without somatic SNP input), and CNAnorm, which relies solely on sequencing depth. Accuracy is evaluated as 1/RMSE, with higher values indicating more precise estimation.


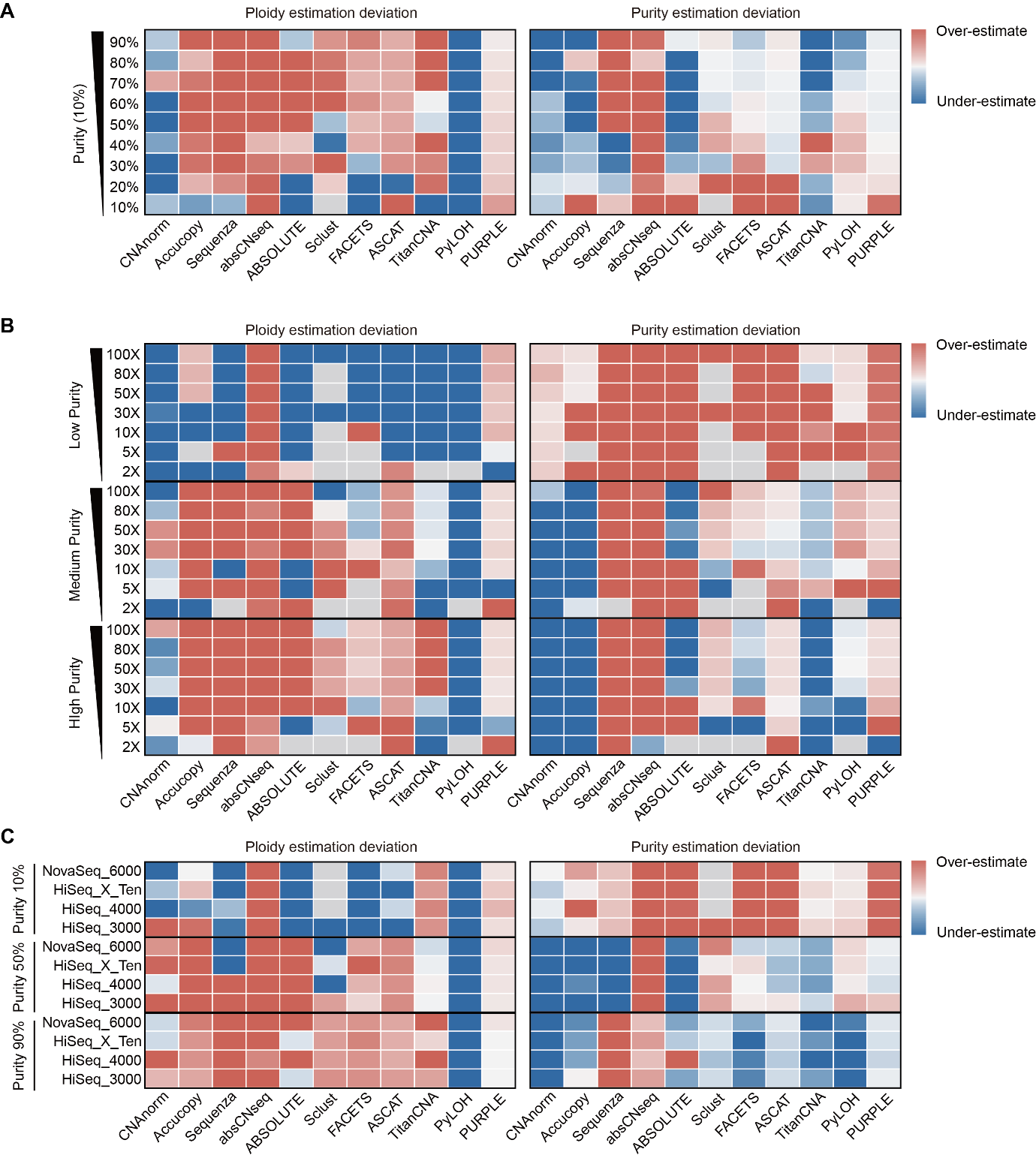
**Figure S3. Estimation bias of bulk analysis tools.**

Bias in ploidy (left) and purity (right) estimation by bulk tools for Dataset 3 (A), Dataset 4 (B), and Dataset 2 (C). Blue indicates under-estimation, red indicates over-estimation, with darker color intensity representing larger deviations from ground truth. White denotes no discrepancy between estimated and true values.


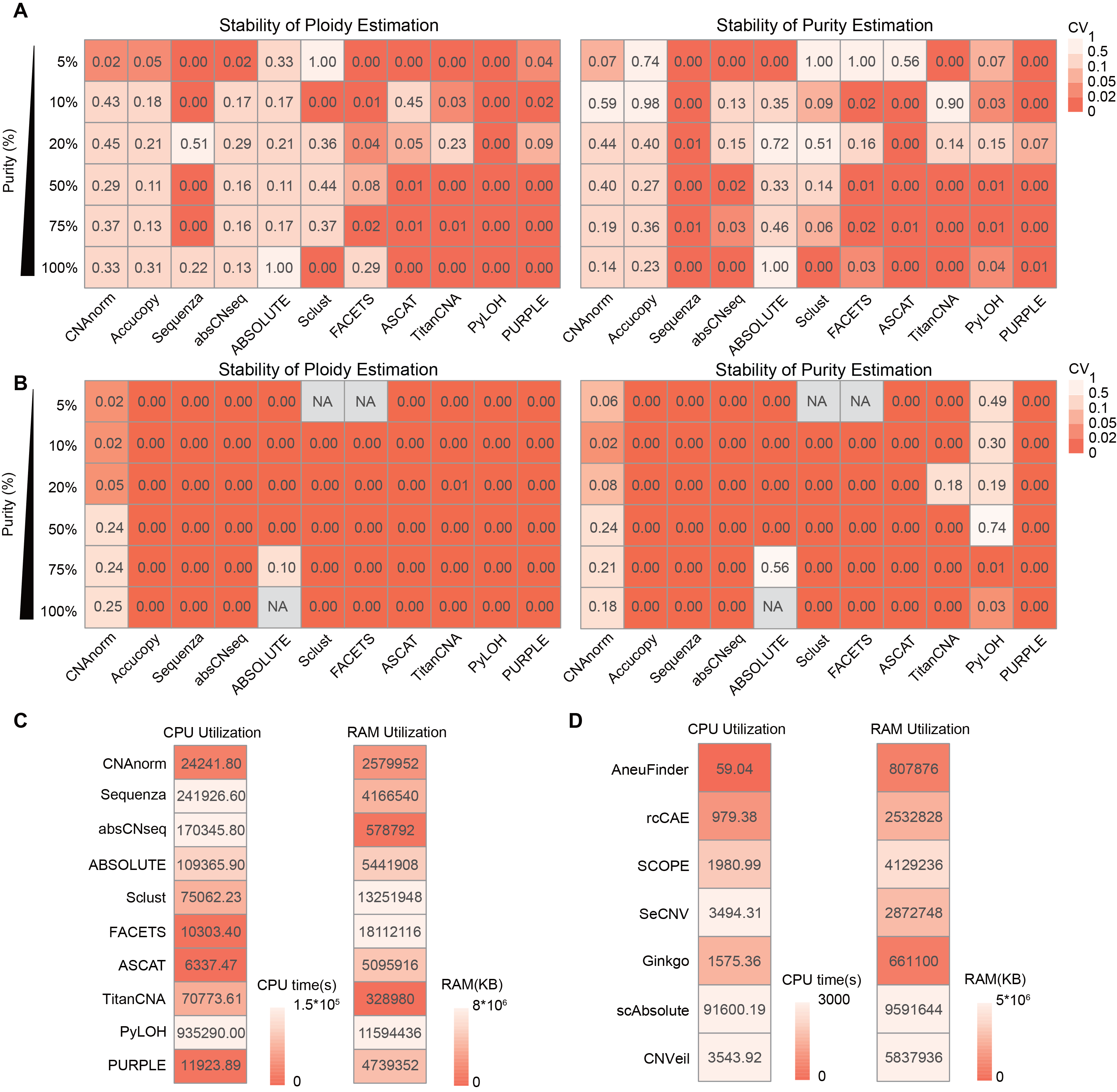
**Figure S4. Stability and computational performance of the evaluated tools.**

(A, B) Stability of ploidy and purity estimation across three technical replicates (A) and 5 repeated runs (B) for different tools, assessed using samples with varying purity levels (5% to 100%) from Dataset 1. Stability was quantified using the coefficient of variation (CV), with warmer/darker colors indicating higher stability. (C, D) Computational performance of bulk (C) and single-cell (D) methods, including CPU time (left) and memory (RAM) usage (right). All metrics were obtained using the GNU `time` utility.

**Table S1.** Datasets used in this study.

| Datasets | | Purity | Ploidy | Read length | Genome coverage (bulk) /  Cell number (scDNA-seq) | Platform | Experimental (E)/ simulated (S) | Accession number |
| --- | --- | --- | --- | --- | --- | --- | --- | --- |
| Bulk WGS | Dataset 1 | 0%, 5%, 10%, 20%  50%, 75%, 100% | 2.8N | 150 bp × 2 | ~140X | HiSeq 4000 | E | PRJNA489865 |
|  | Dataset 2 | 50% | 4N, 8N | 151bp x 2 | ~15X | DNBSEQ-T7 | E | PRJNA1256151 |
|  | Dataset 3 | 10%, 50%, 90% | 2.8N | 150 bp × 2 (short read)  3.3 Kb (Nanopore)  5.2 Kb (PacBio) | ~57X  ~89X  ~42X  ~68X  ~11X  ~40X | HiSeq 3000  HiSeq 4000  HiSeq X Ten  NovaSeq 6000 Nanopore  PacBio | S | Simulated from PRJNA489865 |
|  | Dataset 4 | 10%, 20%, 30%, 40%, 50%, 60%, 70%, 80%, 90% | 2.8N | 150 bp × 2 | ~140X | HiSeq 4000 |  |  |
|  | Dataset 5 | 10%, 50%, 75% | 2.8N | 150 bp × 2 | 2X, 5X, 10X, 30X, 50X, 80X, 100X | HiSeq 4000 |  |  |
| Single-cell DNA-seq | ACT dataset | / | 3.45N, 3.03N, 3.44N, 3.76N, 2.65N, 3.17N, 3.15N, 3.95N | 50 bp, 76bp | 9106 cells | HiSeq 4000 | E | PRJNA629885 |
|  | scWGA dataset | / | 1.7N, 2N, 3N, 3.3N | 48 bp, 76 bp | 99 cells | HiSeq2000 | E | SRX021401 |

**Table S2.** A brief summary of ploidy estimation methods for bulk WGS

| Tools | Mode | Input | Version | Algorithm | Data type |  | Call ASCN | Segmentation algorithm | Year |
| --- | --- | --- | --- | --- | --- | --- | --- | --- | --- |
| ASCAT | Tumor-normal paired/Tumor-only | Bam | 3.2.0 | Read depth & BAF | WGS/WES  Long-read-sequence |  | Y | Allele-Specific Piecewise Constant Fitting (PCF) | 2010 |
| CNAnorm | Tumor-normal paired | Bam | 1.48.0 | Read depth | WGS |  | N | Circular binary segmentation (CBS) | 2011 |
| ABSOLUTE | Tumor-normal paired | Segmentation file &  SNP file(optional) | 1.0.6 | Read depth & BAF | WGS |  | Y | Tool lacks built-in segmentation; GATK's multidimensional kernel segmentation + MCMC smoothing was used | 2012 |
| absCNAseq | Tumor-normal paired | Bam & SNP file | 1.0 | Read depth & BAF | WGS/WES |  | Y | Circular binary segmentation (CBS) | 2013 |
| PyLOH | Tumor-normal paired | Bam &  Segmentation file(optional) | 1.4.3 | Read depth & BAF | WGS/WES |  | Y | Tool lacks built-in segmentation; GATK's multidimensional kernel segmentation + MCMC smoothing was used | 2014 |
| TITAN | Tumor-normal paired | Bam | 1.44.0 | Read depth & BAF | WGS/WES |  | N | Hidden Markov Model (HMM) | 2014 |
| Sequenza | Tumor-normal paired | Bam | 3.0.0 | Read depth & BAF | WGS/WES |  | Y | Piecewise constant fit (PCF) | 2015 |
| FACETS | Tumor-normal paired | Bam | 0.16.1 | Read depth & BAF | WGS/WES |  | Y | Bivariate Hotelling T2 statistic (extended from CBS) | 2016 |
| Sclust | Tumor-normal paired | Bam & SNP file | 1.1 | Read depth & BAF | WGS/WES |  | Y | Circular binary segmentation (CBS) | 2018 |
| PURPLE | Tumor-normal paired/Tumor-only | Bam | 3.8.4 | Read depth & BAF | WGS |  | Y | Piecewise constant fit (PCF) | 2019 |
| Accucopy | Tumor-normal paired | Bam | debug | Read depth & BAF | WGS |  | Y | Sparse-Bayesian-Learning based segmentation | 2021 |

**Table S3.** A brief summary of ploidy estimation methods for scDNA-seq

| Tools | Mode | Input | Output | Version | Single (S) or multiple (M) samples | Mappability correction | Outlier cell removal | Segmentation algorithm | Year |
| --- | --- | --- | --- | --- | --- | --- | --- | --- | --- |
| HMMcopy | Tumor-only | Bam | 1. Ploidy  2. CNV profile | 1.44.0 | S | Y | N | Hidden Markov Model (HMM) | 2006 |
| Ginkgo | Tumor-only | Bam | CNV profile | Default | S | N | Y | Circular binary segmentation (CBS) | 2015 |
| AneuFinder | Tumor-only | Bam | CNV profile | 1.34.0 | S | N | Y | Hidden Markov Model (HMM) | 2016 |
| SCOPE | Tumor-normal paired | Bam | 1. Ploidy  2. CNV profile | 3.20 | M | Y | Y | Generalized likelihood ratio test to jointly segment all cells; Modified Bayesian information criterion (BIC) to determine the optimal number of segments | 2020 |
| SeCNV | Tumor-only | Bam | 1. Ploidy  2. CNV profile | 0.1.1 | M | Y | Y | Minimizing the structural entropy from a depth congruent map | 2022 |
| rcCAE | Tumor-only | Bam | 1. Ploidy  2. CNV profile | Default | M | Y | Y | Hidden Markov Model (HMM) | 2023 |
| CNVeil | Tumor-normal paired | Bam | 1. Ploidy  2. CNV profile | Default | M | Y | Y | Change rate-based across-cell breakpoint identification approach | 2024  (bioRxiv) |
| scAbsolute | Tumor-only | Bam | Ploidy | Default | S | Y | N | PELT algorithm for change point detection with a negative binomial likelihood to find an initial segmentation | 2024 |

| Sample | Ploidy | N | %outlier(mean distance) | | | | | | | |
| --- | --- | --- | --- | --- | --- | --- | --- | --- | --- | --- |
|  |  |  | AneuFinder | CNVeil | Ginkgo | HMMcopy | SCOPE | SeCNV | rcCAE | scAbsolute |
| TN1 | 3.45 | 1100 | 89.0(0.28) |  | 10.3(0.16) | 5.7(0.11) |  | 6.6(0.09) | 9.1(0.24) | 4.1(0.06) |
| TN2 | 3.03 | 1024 | 65.82(0.23) |  | 8.6(0.09) | 11.1(0.16) |  | 11.4(0.11) | 2.5(0.11) | 9.3(0.10) |
| TN3 | 3.44 | 1101 | 93.82(0.32) |  | 21.9(0.17) | 41.0(0.99) |  | 7.8(0.10) | 6.3(0.14) | 9.7(0.16) |
| TN4 | 3.76 | 1307 | 93.96(0.66) |  | 92.0(1.27) | 44.3(0.42) |  | 38.2(0.62) | 100.0(1.66) | 30.5(0.41) |
| TN5 | 2.65 | 1238 | 92.81(0.48) |  | 11.2(0.25) | 42.7(1.08) |  | 7.7(0.18) | 2.2(0.14) | 18.8(0.47) |
| TN6 | 3.17 | 1205 | 93.44(0.33) |  | 86.3(1.16) | 7.1(0.17) |  | 1.5(0.10) | 11.6(0.12) | 2.2(0.10) |
| TN7 | 3.15 | 907 | 96.25(0.35) |  | 99.3(0.66) | 15.4(0.16) |  | 5.8(0.10) | 5.9(0.13) | 8.9(0.16) |
| TN8 | 3.95 | 1224 | 99.67(0.38) |  | 92.9(0.69) | 21.5(0.15) |  | 3.1(0.08) | 54.6(0.19) | 4.5(0.13) |
| Mean |  |  | 91.0(0.39) |  | 53.4(0.58) | 24.4(0.42) |  | 10.8(0.18) | 26.2(0.37) | 11.4(0.21) |
| H | 1.7 | 24 | 0.0(0.14) | 0.0(0.00) | 0.0(0.02) | 4.2(0.11) | 0.0(0.02) | 0.0(0.00) | 0.0(0.08) | 0.0(0.02) |
| D | 2 | 47 | 8.5(0.20) | 4.3(0.02) | 8.5(0.11) | 17.0(0.43) | 6.4(0.07) | 6.4(0.08) | 4.3(0.10) | 6.4(0.05) |
| AA | 3 | 24 | 45.8(0.23) | 0.0(0.10) | 12.5(0.19) | 12.5(0.35) | 8.3(0.13) | 4.2(0.12) | 4.2(0.13) | 4.2(0.15) |
| AB | 3.3 | 4 | 100.0(0.68) | 100.0(0.45) | 100.0(0.50) | 100.0(0.58) | 100.0(0.46) | 100.0(0.50) | 100.0(0.88) | 100.0(0.47) |
| Mean |  |  | 90.2(0.21) | 17.2(0.05) | 52.8(0.12) | 24.4(0.34) | 9.5(0.09) | 10.8(0.09) | 26.0(0.13) | 11.4(0.08) |

**Table S4.** Outlier analysis and prediction deviation in ploidy estimation across single-cell sequencing methods
